# Is decompression in acute thoracolumbar intervertebral disc herniation overvalued?

**DOI:** 10.3389/fvets.2022.1049366

**Published:** 2022-11-09

**Authors:** Paul Freeman, Nick Jeffery

**Affiliations:** ^1^Department of Veterinary Medicine, University of Cambridge, Cambridge, United Kingdom; ^2^Department of Small Animal Clinical Studies, College of Veterinary Medicine and Biomedical Sciences, Texas A&M University, College Station, TX, United States

**Keywords:** hemilaminectomy, dog, recovery, disc, decompressive

## Introduction

The syndrome later recognized to be caused by acute intervertebral disc herniation in dogs was first described in the late 1800s [report by Dexler [1896] cited in (1)] and characterized in a full pathological description by Hansen in 1952 (2). Acute disc herniation has thus been recognized as a cause of myelopathy and pain for many decades, but controversy continues regarding the most appropriate therapy and its timing.

Pathological studies clearly indicate a spectrum of associated mechanisms of spinal cord injury, ranging from almost pure contusion with minimal spinal cord compression, to highly compressive lesions (2). In each individual, the proportion of tissue damage caused by compressive and contusive mechanisms can be subjectively estimated from magnetic resonance images but cannot be easily quantified. The distinction is important because if the cause is mainly compression then there may clearly be a role for decompressive surgery, but if the primary destructive mechanism is largely contusive, traditional decompressive surgery may have little value unless the meninges are incised, a procedure which itself currently has uncertain benefit (3–6).

## History of spinal decompression for herniated disk

Historically, affected dogs were treated conservatively, by simply waiting for recovery, or given a variety of unproven medical therapies [such as vitamin B1 injections (1)]. During the 1950s and 1960s various types of surgery, including dorsal laminectomy, hemilaminectomy and disc fenestration were introduced and thought to provide additional benefit (1, 7–9) although it is important to note that these techniques have never been formally analyzed for efficacy *versus* conservative therapy. During the 1970s-1990s multiple disc fenestrations was widely used as a sole therapy, with reported high success rates, for treatment of all grades of loss of neurologic function associated with thoracolumbar disc herniation (10–14). However, in the 1990s this approach was largely abandoned in favor of “decompressive” surgery, predominantly hemilaminectomy (15–17), possibly in part due to the increased availability of cross-sectional imaging that revealed distortion of spinal cord shape and dimensions that previously had been difficult to appreciate, frequently leading to a diagnosis of spinal cord compression (18). The degree of compression that is clinically meaningful in this context has never been formalized; indeed, it may well be impossible to define reliable boundary values because of the individual variability in mix of contusive and compressive mechanisms of injury. Regardless, case series consistently report good outcomes following decompressive surgery, with recovery rates for dogs with intact deep pain perception varying between 63 and 100%, but mostly around 90% (16, 19–24).

Nevertheless, summary recovery rates for dogs treated by decompressive surgery or by fenestration alone are indistinguishable (23) and, importantly, there have been no clinical trials comparing decompression with non-surgical treatment or fenestration. Despite this lack of evidence a dogma has developed that decompressive surgery is superior and perhaps even necessary for recovery (25). Therefore, at present, the consensus of opinion is that dogs that are unable to walk following acute onset thoracolumbar disc herniation are candidates for decompressive surgery—overwhelmingly using the 70-year-old technique described by Greene (7), often with little modification from the original description.

## Could the current consensus be erroneous?

It cannot be argued that the current consensus therapy does not produce good results, given 90–95% of deep pain positive dogs recover to walk again after decompressive surgery (23, 24). However, it is possible that the good outcomes that are almost invariably attained, independent of the degree of compression or the amount of disc material left in the canal (26), do not represent a response to surgery, but are simply the natural history of the condition. This remains a tenable argument until the value of decompressive surgery is proven in randomized controlled trials, and is supported by the equally excellent outcomes following fenestration alone—a procedure that does not decompress the spinal cord.

### Controversy regarding the importance of spinal cord compression

It is known that there is poor correlation between spinal cord compression and severity of neurological deficits at presentation or probability of recovery (27), assumed to be because of the interacting, and possibly more dominant, effect of spinal cord contusion. There is evidence from observational studies both for and against the importance of spinal cord compression as a cause of injury and therefore an indication for decompressive surgery.

#### Lines of evidence supporting the importance of compression (and therefore decompressive surgery)

1. Compression associated with disc herniation is self-evident on imaging and can appear very severe [although see (28)].

2. High recovery rates following decompressive surgery in numerous case series. In addition, individual cases anecdotally [and are also reported in some series *e.g.*, see (29)] show spectacularly rapid improvement in neurologic function after decompression, implying that for some herniations decompression is likely highly valuable.

3. The often-repeated claim that dogs recover more rapidly and more completely following decompressive surgery and that there are fewer recurrences (30). However, there are no formal comparison studies to support any of these assertions and even a suggestion of an increased risk of recurrence following dorsal laminectomy (31). A systematic review (24) concluded that there was a trend for a higher proportion of recovery and more rapid recovery in non-ambulatory dogs undergoing hemilaminectomy than conservative treatment. On the other hand, the authors also cautioned that those inferences were based on low level evidence that included a high risk of selection and other biases.

4. The apparently higher proportion of deep pain negative dogs recovering following decompressive surgery than with conservative management (24) although, again, this is based on small case numbers and limited follow-up.

#### Lines of evidence supporting the unimportance of spinal cord compression following thoracolumbar disc herniation

1. Recovery of a large proportion of dogs following conservative therapy. A problem with interpreting reports on conservative therapy is that many dogs were ambulatory at the onset of treatment and so might be considered more likely to recover; on the other hand, they also have more scope to deteriorate. More crucially, many reported as “failing to recover” may still have recovered with conservative or rehabilitative therapy alone if allowed more time, but were diverted to decompressive surgery at that stage (32–35).

2. The very similar proportions of cases recovering with fenestration alone (85–100% deep pain positive dogs) as following decompressive surgery. An unsubstantiated argument put forward for a benefit of fenestration is that it might reduce “dynamic compression” associated with disc herniation (1).

3. The equivocal severity of compression of the spinal cord observed in many, perhaps the majority of, clinical cases. In an experimental study examining the effects of combined contusion and compression in rats, spinal cord compression of < 50% had minimal impact on timing of recovery of ambulation (36).

4. The documented possibility of spontaneous recovery of function and amelioration of compression, even if severe, in at least some cases (28, 37).

## Why does it matter?

There is a widespread belief, supported by cursory scanning of social media, amongst many dog owners and primary care veterinarians that acute thoracolumbar disc herniation not only necessitates surgery but requires *emergency* surgery. The primary reason for considering these cases emergencies centers on the concern that the neurologic signs may progress, possibly to a non-recoverable status. The proportion of deep pain negative dogs that will recover ambulation following surgery is only between 55–60% (23, 24), much less than that expected in dogs that retain deep pain perception. There is therefore, not unreasonably, a fear that surgical delay will “allow” dogs to deteriorate to become deep pain negative, at which point the prognosis for recovery is assumed to drop precipitously.

Therefore, at present, many owners are confronted with a perceived requirement for their dog to undergo an immediate and costly surgical procedure to prevent permanent paralysis. When put on the spot in this way many owners with limited financial resources may opt for euthanasia, apparently sometimes supported by their primary care veterinarian, so as to spare their pet the possibility of unnecessary suffering.

## Timing of surgery

In view of the known results of non-surgical management, euthanasia is clearly not an appropriate choice for owners that cannot afford decompressive surgery. The dilemma is further exacerbated by the mixed evidence on whether decompressive surgery should be considered an emergency. If it were possible to delay surgery it would become affordable for many more owners.

For deep pain negative dogs there is already evidence that the time elapsed in this status does not affect recovery of ambulation following surgery (29, 38) but much less is known regarding those that retain pain perception. While it is well recognized that some dogs will deteriorate from deep pain positive (DPP) to deep pain negative (DPN), the proportion in which this will occur, and the timescale over which it happens, are both highly uncertain. Given the overall good recovery rates for dogs that retain pain perception, it is surprising that the more important clinical issue of pre-operative deterioration from DPP to DPN has rarely been examined. Martin et al. (39) reported that a greater proportion of dogs lost deep pain perception overnight if unoperated when compared to dogs operated the same day, suggesting the need for early surgery.

However, in contrast, in the series reported by Rosen et al. (40) only one dog of a total of 75 DPP at presentation deteriorated to become DPN before surgery (during an 8-h delay to surgery; many dogs had much longer delays) and one dog deteriorated from DPP before surgery to become DPN after surgery, thereby suggesting no need for surgical urgency.

Overall, there is currently not unequivocal evidence to suggest that operating on a DPP dog is urgent although, on the other hand, there is no suggestion that it is detrimental either—simply strong evidence that it is not warranted in ALL patients. There is also plentiful evidence from human and canine studies that cord compression associated with a herniated disc can, at least in some cases, disappear without medical intervention (28, 37, 41–43). Anecdotally we know that some cases appear to respond rapidly to surgical decompression, whereas many do not. Therefore, the key question is whether it is possible to identify which cases require decompression, or urgent decompression, and which do not. A possible solution is that if dogs could be treated conservatively until a specific period has elapsed—perhaps ~3-4 weeks—when most operated dogs would be expected to recover ambulation (40) then those that haven't recovered at that time would be considered strong candidates for decompressive surgery.

## Where do we go from here?

The general acceptance that surgical decompression is necessary for recovery, especially in dogs that lose deep pain sensation, makes constructing clinical trials of alternative approaches highly problematic. Indeed, in the current opinion environment (25), it would be difficult for any specialist neurologist NOT to recommend decompressive surgery for a non-ambulatory dog with a herniated thoracolumbar disc. So, in view of this ethical background, how can this potentially fallacious dogma be challenged? Several pathways are possible, but an important line of data would be outcomes of dogs that are clear candidates for decompressive surgery according to the current consensus, given their presentation and imaging, but in which this therapy is unavailable because of cost or accessibility. If the outcomes were broadly comparable with those associated with decompressive surgery it would open the path toward randomized controlled trials to compare conservative and surgical therapies, thereby rectifying the current deficiency in formal testing. A greater accumulation of imaging and outcome data following conservative therapy might also aid in suggesting factors associated with poor recovery and allow more rational allocation of animals to surgical or conservative therapy in future.

## Author contributions

All authors listed have made a substantial, direct, and intellectual contribution to the work and approved it for publication.

## Conflict of interest

The authors declare that the research was conducted in the absence of any commercial or financial relationships that could be construed as a potential conflict of interest.

## Publisher's note

All claims expressed in this article are solely those of the authors and do not necessarily represent those of their affiliated organizations, or those of the publisher, the editors and the reviewers. Any product that may be evaluated in this article, or claim that may be made by its manufacturer, is not guaranteed or endorsed by the publisher.
